# Genetic and functional evidence for a locus controlling otitis media at chromosome 10q26.3

**DOI:** 10.1186/1471-2350-15-18

**Published:** 2014-02-06

**Authors:** Marie S Rye, Elizabeth SH Scaman, Ruth B Thornton, Shyan Vijayasekaran, Harvey L Coates, Richard W Francis, Craig E Pennell, Jenefer M Blackwell, Sarra E Jamieson

**Affiliations:** 1 Telethon Institute for Child Health Research, The University of Western Australia, Perth, Western Australia, Australia; 2 School of Paediatrics and Child Health, University of Western Australia, Perth, Australia; 3 School of Women’s and Infants’ Health, University of Western Australia, Perth, Australia; 4 Department of Otolaryngology, Head and Neck Surgery, Princess Margaret Hospital for Children, Perth, Australia; 5 Department of Otolaryngology, Head and Neck Surgery, University of Western Australia, Perth, Western Australia, Australia

**Keywords:** Acute otitis media, Otitis media with effusion, Genetic polymorphisms, Linkage, Association, Raine study, WAFSOM, Australia

## Abstract

**Background:**

Otitis media (OM) is a common childhood disease characterised by middle ear effusion and inflammation. Susceptibility to recurrent acute OM and chronic OM with effusion is 40-70% heritable. Linkage studies provide evidence for multiple putative OM susceptibility loci. This study attempts to replicate these linkages in a Western Australian (WA) population, and to identify the etiological gene(s) in a replicated region.

**Methods:**

Microsatellites were genotyped in 468 individuals from 101 multicase families (208 OM cases) from the WA Family Study of OM (WAFSOM) and non-parametric linkage analysis carried out in ALLEGRO. Association mapping utilized dense single nucleotide polymorphism (SNP) data extracted from Illumina 660 W-Quad analysis of 256 OM cases and 575 controls from the WA Pregnancy Cohort (Raine) Study. Logistic regression analysis was undertaken in ProbABEL. RT-PCR was used to compare gene expression in paired adenoid and tonsil samples, and in epithelial and macrophage cell lines. Comparative genomics methods were used to identify putative regulatory elements and transcription factor binding sites potentially affected by associated SNPs.

**Results:**

Evidence for linkage was observed at 10q26.3 (Z_lr_ = 2.69; P = 0.0036; D10S1770) with borderline evidence for linkage at 10q22.3 (Z_lr_ = 1.64; P = 0.05; D10S206). No evidence for linkage was seen at 3p25.3, 17q12, or 19q13.43. Peak association at 10q26.3 was in the intergenic region between *TCERG1L* and *PPP2R2D* (rs7922424; P = 9.47 × 10^-6^), immediately under the peak of linkage. Independent associations were observed at *DOCK1* (rs9418832; P = 7.48 × 10^-5^) and *ADAM12* (rs7902734; P = 8.04 × 10^-4^). RT-PCR analysis confirmed expression of all 4 genes in adenoid samples. *ADAM12*, *DOCK1* and *PPP2R2D*, but not *TCERG1L*, were expressed in respiratory epithelial and macrophage cell lines. A significantly associated polymorphism (rs7087384) in strong LD with the top SNP (rs7922424; r^2^ = 0.97) alters a transcription factor binding site (CREB/CREBP) in the intergenic region between *TCERG1L* and *PPP2R2D*.

**Conclusions:**

OM linkage was replicated at 10q26.3. Whilst multiple genes could contribute to this linkage, the weight of evidence supports *PPP2R2D,* a TGF-β/Activin/Nodal pathway modulator, as the more likely functional candidate lying immediately under the linkage peak for OM susceptibility at chromosome 10q26.3.

## Background

Otitis media (OM) is a common childhood disease characterised by the presence of infection (acute otitis media; AOM) or fluid (otitis media with effusion; OME) in the middle ear cavity. Most children experience at least one episode of AOM by 1 year of age with up to 40% experiencing recurrent episodes of AOM (rAOM; ≥3 episodes of AOM in 6 months) or chronic episodes of OME (COME; middle ear effusion (MEE) ≥3 months) in childhood [1]. Recurrent disease can result in perforation of the tympanic membrane and/or conductive hearing loss, leading to deficits in language development and poor educational outcomes. Treatment for recurrent disease may include insertion of tympanostomy tubes. High prevalence rates of OM result in substantial health care related costs and significant childhood morbidity in many countries [2].

The causal mechanisms that lead to recurrent disease are poorly understood but familial clustering and high heritability estimates point to a genetic component. Using self-reported AOM history from 2,570 Norwegian twin pairs, Kvaerner *et al*. [3] determined heritability estimates of 0.45 in males and 0.74 in females. Two prospective twin and triplet studies have confirmed these estimates. Casselbrant *et al.*[4] reported heritability estimates of 0.73 for time with MEE in the first two years of life. Likewise, Rovers *et al.*[5] reported heritability estimates of 0.49 at 2 years increasing to 0.71 at 4 years with a concomitant decrease in shared environment estimates from 0.41 at 2 years to 0.16 by 4 years of age. Overall these epidemiological studies confirm that susceptibility to OM has a substantial genetic component that increases with age.

To date, there have been few studies undertaken to pinpoint the genes involved. Two genome-wide linkage scans using multi-case families of Caucasian origin have identified specific regions of the genome harbouring putative susceptibility genes. The first identified two regions of linkage on chromosome 10q26.3 and 19q13.43, and a further region on 3p25.3 after conditioning on the linked regions, suggesting epistatic interactions [6]. The second identified two different regions of linkage on 17q12 and 10q22.3 [7]. Whilst the region of 19q13.43 has since been refined [8], there has been no replication of any of these regions in independent studies and the causal genes underlying them have yet to be identified.

To determine whether these genomic regions are important in recurrent/severe OM in a Western Australian population we have carried out linkage analysis using families who contain at least two individuals diagnosed with rAOM or chronic OME (COME) recruited to the Western Australian Family Study of Otitis Media (WAFSOM) [9]. To identify the putative disease susceptibility locus in the only region that replicated in this population, association using SNPs was undertaken in the Western Australian Pregnancy Cohort (Raine) Study [10]. Functional and bioinformatic studies were used to further clarify the putative etiological gene.

## Methods

### Sample collection and phenotype definition

Linkage studies utilised samples from the WAFSOM where probands with a history of tympanostomy tube insertion due to rAOM or COME were identified from the records of collaborating ear, nose and throat (ENT) specialists (HC and SV) as previously described [9]. Parents and full siblings with a history of recurrent disease, defined as ≥3 physician diagnosed episodes of AOM or tympanostomy tube insertion for rAOM or COME, were also invited to participate. No exclusion was made on the basis of ethnicity or gender; 93.2% of the families in the WAFSOM self-identified as Caucasian. Recruitment to the WAFSOM was approved by the Human Ethics Committee at Princess Margaret Hospital for Children (PMH). Written, informed consents were obtained both for participation in the study and for DNA collection from all adults or from the parents of participants less than 18 years of age. For linkage analysis family members with no history of recurrent disease were classified as unaffected whilst all others were classified as unknown. A total of 101 multicase families (107 nuclear families; 208 affected individuals; 468 total individuals) with 2 to 4 affected individuals were included in the linkage analysis.

For association mapping, we utilised data from the Western Australian Pregnancy Cohort (Raine) Study (‘the Raine Study’), a longitudinal cohort of children whose mothers were recruited during early pregnancy [10]. For the purposes of this study, data collected from clinical examinations and parental questionnaires completed each year at the (average) ages of one, two and three years was used to define a phenotype for OM. Children were defined as a case if clinical examination in the first three years of life indicated presence of inflamed, retracted or scarred TM, MEE or tympanostomy tubes *in situ*. Participants were also classified as a case where parents’ yearly reports indicated ≥3 episodes of AOM had occurred up to the age of 3 yrs; 35% of cases qualified on the basis of yearly questionnaire criteria alone. Children with no clinical or parental reported history of OM by the age of 3 yrs were classified as controls. Based on questionnaire data, 94% of the Raine Study participants self-identified their ethnicity as Caucasian. We used a subset of 831 Raine Study participants (256 cases and 575 controls) for whom both genome-wide data from an Illumina 660 W Quad Beadchip and complete epidemiological data for covariates were available [11]. Covariates included day care attendance at <3 years of age, allergy diagnosed at <3 years of age, and non-exclusive breastfeeding from <6 months of age, all of which were significant risk factors for OM in the Raine Study cohort [11]. Recruitment to the Raine Study and all follow-ups were approved by the Human Ethics Committee at King Edward Memorial Hospital and/or PMH, with specific adult re-consent for DNA for those individuals participating in this study.

### DNA extraction

High molecular weight genomic DNA was extracted from a 2 mL saliva sample using the Oragene technology (DNA Genotek) as per manufacturer’s instructions for the WAFSOM study. Extracted DNA was re-suspended in TE buffer, quantified by spectrophotometry, and stored at 50 ng/μl at -20C.

For the majority of Raine Study participants genomic DNA (gDNA) was extracted from whole blood collected at the 14- or 17-year follow-up via venipuncture utilising 4 mL K_2_EDTA vacuum tubes. Extraction of DNA from whole blood was performed utilising Qiagen PureGene chemistry. For a small subset of individuals (~5%), gDNA was extracted from saliva using the Oragene technology (DNA Genotek) as per manufacturer’s instructions. Samples were quantified using spectrophotometry, diluted to a normalised concentration with reduced EDTA TE buffer and stored at −80°C.

### Marker selection

For linkage analysis a minimum of four microsatellite markers [12] spanning each reported [6,7] region of linkage were identified. Marker names and primer sequences are provided in Additional file 1: Table S1. For 10q26.3, 19q13.43 and 3p25.3 the reported marker at the peak of linkage [6] was chosen in addition to four flanking markers. Association mapping across the 10q26.3 region was undertaken in the Raine Study cohort by extracting data for 10,185 SNPs (2,270 genotyped, 7,915 by imputation [13]) from cleaned [14], imputed [13] Illumina 660 W-Quad Beadchip data for 831 individuals (256 cases and 575 controls).

### Genotyping

Microsatellite PCRs were performed on WAFSOM DNAs using 15 ng genomic DNA, 1X PCR buffer, 2.6 mM MgCl_2_, 0.45 mM dNTPs, 0.04 M betaine, 0.87 μM - 1.1 μM forward and reverse primers, and 0.25U of AmpliTaq Polymerase. Cycling was performed using a touchdown protocol [15]. Amplification products were pooled, fragments resolved using an Applied Biosystems 3130xl Genetic Analyzer (Life Technologies, California) and alleles assigned via GeneMapper v4.1. All microsatellites were in Hardy-Weinberg equilibrium in genetically unrelated founders. Mendelian inconsistencies were identified using the Pedcheck software [16] and corrected or removed prior to analysis.

Genotype data for the Raine Study was generated at the Centre for Applied Genomics (Toronto, Canada) using an Illumina 660 W Quad Beadchip (Illumina, San Diego, California). Quality control (QC) and imputation for the Raine Study has previously been described [17]. Briefly, QC checks were performed for individuals and SNPs using PLINK [18]. Individuals were excluded based on gender mismatch, low genotyping rate (<97%), related to other participants or low level of heterozygosity. SNPs were excluded on the basis of deviation from Hardy-Weinberg Equilibrium (HWE P < 5.7 × 10^-7^), a genotype call rate <95%, or minor allele frequency <1%. For the chromosome 10q26.3 region, a total of 2,270 genotyped SNPs passed QC checks. Imputation was performed using MACH v1.0.16 with CEU samples from the HapMap Phase2 (Build 36, release 22) used as a reference panel and a default threshold of r^2^ > 0.3. After imputation, 10,185 SNPs (2,270 genotyped, 7,915 imputed) were available for analysis.

### Linkage and association analysis

For microsatellite markers single-point and multi-point non-parametric linkage (NPL) analysis was carried out using all 101 multicase families with the S_all_ scoring function in the program ALLEGRO [19] with genetic map distances obtained from the Rutgers Map v2 [12]. Conditional linkage analysis was also performed in ALLEGRO using only families showing evidence for linkage (Z_lr_ >0) at a specified region.

Association mapping of the 10q26 region was performed using ProbABEL [20] for 10,185 SNPs in the region 127 Mb to qter. Power approximations [21] estimated for a disease prevalence of 0.4 show that the Raine Study has 86% power to detect associations at an alpha level of P = 1 × 10^-5^ with genotype relative risk (GRR) of 1.5 for SNPs with a MAF = 0.2 and 95% power at P = 1 × 10^-4^. Analysis was initially performed adjusting for population substructure (i.e. including the first two Principal Components; PCs), and then repeated, where appropriate, adjusting for PCs and independently associated covariates. If a strict Bonferroni correction was applied then the P-value required to correct for the number of SNPs analysed in the Raine Study data is P = 4.9 × 10^-6^ (P = 0.05/10,185). However, this is likely to be highly conservative due to the known presence of linkage disequilibrium (LD) in this region (i.e. 629 LD blocks across this 10q26 region, data not shown). Therefore, as this is a replicated linkage region [6,7], a relaxed threshold of P < 10^-3^ was used to identify SNPs/genes of interest for follow-up. Logistic regression modelling for independent effects between pairs of SNPs was undertaken in R version 2.15.0 [22], and results presented as P_LRT_ for the likelihood ratio test comparison. Regional plots of association were created using LocusZoom [23] in which -log_10_(P values) were graphed against their chromosomal location. Pairwise LD patterns between all regional SNPs and the respective top SNP were calculated using the HapMap CEU population.

### Qualitative PCR expression

For qualitative RT-PCR, RNA was extracted from mononuclear cells isolated from 12 paired adenoid and tonsil samples collected with parent/guardian consent during adenoidectomy and/or tonsillectomy using TRI Reagent (Sigma-Aldrich) according to manufacturer’s instructions [24]. Ethical approval for this part of the study was obtained from the Ethics Committee of PMH. RT-PCR was also performed on two respiratory epithelial and three macrophage cell lines with/without infection with otopathogens. All reagents were from Life Technologies, Australia unless otherwise stated. Adenocarcinomic alveolar basal epithelial cells (A549) were cultured in DMEM, 10% fetal bovine serum (FBS), 2 mM L-glutamine and 100U/ml penicillin/100 μg/ml streptomycin. A pharyngeal carcinoma epithelial cell line (Detroit 562) was grown in MEM with Earle’s salts, 10% FBS, 2 mM L-glutamine, 1X non-essential amino acids, 1 mM sodium pyruvate and 100U/ml penicillin/100 μg/ml streptomycin. Both the promonocytic U937 and monocytic THP-1 cell lines were grown in RPMI 1640, 10% FBS, 2 mM L-glutamine and 100U/ml penicillin/100 μg/ml streptomycin whilst the MonoMac 6 (MM6) mature monocytic cell line additionally required 1X non-essential amino acids and 9 μg/ml OPI bovine insulin (Sigma-Aldrich).

Cell lines were stimulated *in vitro* in 6-well plates with either non-typeable *Haemophilus influenzae* (NTHi) or *Streptoccocus pneumoniae* (SP) at a multiplicity of infection of 10:1. Bacterial cultures were kindly provided by Dr Lea-Ann Kirkham (School of Paediatrics and Child Health, University of Western Australia, Perth). Bacteria were streaked and incubated overnight at 37ºC/5% CO2 on agar plates (blood for SP and chocolate for NTHi). A viable count of bacteria was performed prior to infection using a Helber bacterial counting chamber with Thoma ruling (ProScitech). At the 0 hr time-point, 1 ml of bacteria (1 × 10^8^ cfu/ml), or 1 ml of media for control samples, was added to 1 × 10^7^ cells. After one hour media was replaced to remove non-adhered bacteria. Cells were harvested at 0, 1, 3, 6, 12 and 24 hours using TRI Reagent and RNA extracted accordingly.

A standardised 500 ng of extracted RNA was reverse transcribed using the High Capacity cDNA Reverse Transcription Kit (Life Technologies) as per manufacturer’s instructions. For each cell line an RT negative reaction was used as a negative control. Primers were designed across exon-exon boundaries for four candidate genes (Additional file 1: Table S2) under the linkage peak and for the *OAZ1* housekeeping gene [25]. cDNA was amplified using a touchdown PCR protocol [15] in a volume of 11.5 μl consisting of 20 ng cDNA, a PCR master mix containing 0.85X PCR buffer, 2.6 mM MgCl_2_, 0.45 mM dNTPs, 0.04 M betaine and 0.25 units of AmpliTaq Polymerase (Life Technologies) and 8.7 ng primer. Products were visualised on a 1.5% agarose gel stained with ethidium bromide.

### In silico comparative genomics

The presence of conserved non-coding sequences (CNS), which may harbor important regulatory elements, were examined across the *TCERG1L* and *PPP2R2D* genes (± 10 kb flanking sequence) plus the intervening intergenic region. Genomic sequences and associated annotation for human, mouse and rat were exported from Ensembl (Genome Reference Consortium Release 37, Ensembl Release 67) in FASTA and General Feature File (GFF) format, respectively. Global alignment of genomic sequences was performed in Multi-LAGAN [26,27] and the annotated alignment visualized in SynPlot [28]. CNS regions were defined as regions with a nucleotide sequence conservation level of ≥0.6 that had no associated annotation. To search for putative transcription factor binding sites (TFBS) at SNP locations we used AliBaba v2.1 [29], and MatInspector v8.0.5 [30] with a matrix similarity parameter >0.75. We also assessed selected polymorphisms (± 1000 bp flanking sequence) to determine whether they are located within a CpG island, defined using a criteria of C/G content >50%, ratio of observed to expected CpG >0.6 and length >200 bp. Repetitive elements were firstly masked using RepeatMasker [31] following which CpG islands were identified using CpGIsland Searcher [32] and CpGPlot [33]. Pairwise LD between polymorphisms in the Raine Study was calculated using Haploview 4.1 [34].

## Results

### Linkage analysis

Results of linkage analysis using the 101 multi-case families available in WAFSOM are presented in Table 1. Multi-point (peak Z_lr_ = 2.69; P = 0.0036 at D10S1770) and single-point (Z_lr_ = 2.27; P = 0.012 at D10S1770) NPL analysis provided evidence for linkage between markers at chromosome 10q26.3 and OM, contributed to by both the rAOM and COME phenotypes (Table 2). Analysis of Caucasian families alone did not reveal a substantial difference in single-point results at any region (data not shown); therefore, all multi-case families were used in subsequent analyses. Linkage analysis conditional on the linkage at 10q26.3 provided no evidence of epistatic interactions with 19q13.43 or 3p25.3 in this study population (data not shown), as previously reported [6]. Borderline evidence (multi-point Z_lr_ = 1.65; nominal P = 0.05) for replication of linkage was also observed at 10q22.3 whilst no evidence for linkage (Z_lr_ < 1.65; nominal P > 0.05) was observed at 3p25.3, 17q12 or 19q13.43.

**Table 1:** **Results of multi-point and single-point non-parametric linkage analysis in the WAFSOM across chromosomal regions previously**[6,7]**linked to OM**

**Marker name**	**Genetic map location (cM) Marshfield**	**Physical location (Start; Mb) NCBI build 36**	**Single-point Z**_**lr**_	**P value**	**Multi-point Z**_**lr**_	**P value**	**Information content**
***Chromosome 3p25.3***							
D3S1304	22	6.89	0.00	0.500	−0.93	0.175	0.88
D3S3691	29	8.82	−0.24	0.405	−1.15	0.126	0.90
D3S1597	30	9.34	−0.76	0.225	−1.04	0.149	0.92
D3S1263	36	11.49	−0.22	0.412	−1.13	0.130	0.88
D3S1259^a^	37	12.07	−0.14	0.444	−0.87	0.192	0.76
***Chromosome 10q22.3***							
D10S1730	99	78.60	1.56	0.060	1.48	0.070	0.87
D10S206	98	79.45	1.88	**0.031**	1.65	**0.050**	0.83
D10S1677	100	79.88	0.30	0.383	1.40	0.081	0.87
D10S201	102	80.69	1.71	**0.044**	1.46	0.072	0.86
***Chromosome 10q26.3***							
D10S1655	162	130.85	1.52	0.065	1.65	**0.050**	0.85
D10S169	173	132.41	1.30	0.098	2.51	**0.006**	0.91
D10S1770	169	132.57	2.27	**0.012**	2.69	**0.004**	0.92
D10S212^a^	171	134.29	1.66	**0.048**	2.42	**0.008**	0.87
D10S1711	171	135.05	0.90	0.183	2.52	**0.006**	0.85
***Chromosome 17q12***							
D17S1293	56	29.58	0.32	0.373	0.40	0.345	0.87
D17S933	58	30.22	0.36	0.361	0.28	0.391	0.84
D17S927	58	32.08	0.68	0.247	0.46	0.324	0.88
D17S946	60	34.11	1.63	0.052	1.09	0.137	0.84
***Chromosome 19q13.43***							
D19S572	89	58.80	−0.23	0.409	−0.22	0.413	0.81
D19S210	100	61.71	0.01	0.500	−0.85	0.198	0.94
D19S887	100	62.33	−0.60	0.274	−0.13	0.447	0.96
D19S254	101	62.36	0.19	0.425	−0.22	0.414	0.95
D19S214^a^	101	62.47	−1.85	**0.032**	−0.44	0.330	0.95

^a^Marker at peak of linkage reported by Daly *et al*. [6].

**Table 2:** Results of multi-point and single-point non-parametric linkage analysis in the WAFSOM for the 10q26.3 region stratified by rAOM (85 families; 174 affected individuals) and COME (53 families; 115 affected individuals)

**Marker name**	**rAOM**			**COME**		
	**Multi-point Z**_**lr**_	**P value**	**Information content**	**Multi-point Z**_**lr**_	**P value**	**Information content**
D10S1655	1.83	**0.034**	0.84	1.23	0.110	0.89
D10S169	2.44	**0.007**	0.90	1.76	**0.039**	0.93
D10S1770	2.63	**0.004**	0.91	2.00	**0.023**	0.95
D10S212^a^	2.20	**0.014**	0.86	1.76	**0.039**	0.91
D10S1711	2.34	**0.010**	0.84	1.66	**0.048**	0.89

^a^Marker at peak of linkage reported by Daly *et al*. [6].

### Association mapping of the 10q26.3 region in the Raine Study cohort

In our study, the peak of linkage at 10q26.3 is observed at 169 cM (Marshfield Genetic map)/132.57 Mb (NCBI Build 36), with evidence for linkage extending proximally to 130.85 Mb and distally to 135.05 Mb. Daly *et al*. [6] report clear evidence for linkage only at microsatellite marker D10S212, located at 171 cM/134.29 Mb, with no evidence for linkage ~126 Mb (Table 3). Although the disease locus in a linkage study might normally be found immediately under the peak of linkage [35], the true susceptibility gene can be displaced by up to 10 cM (~10 Mb) from the linkage peak, particularly in smaller samples [36,37]. Therefore, we extended our search for genes through association mapping over the region 127 Mb to 135.35 Mb (=qter).

**Table 3:** **Comparison of results from the WAFSOM linkage analysis for chromosome 10q26 with those published by Daly *et al.*** [6]

**Marker name**	**Genetic map position (cM) Marshfield**	**Physical location (Start; Mb) build 36**	**Spt Z**_**lr**_	**P Value**	**Mpt Z**_**lr**_	**P Value**	**Daly LOD (Spt)**	**Daly LOD (Mpt)**	**Nearest genes Proximal_Distal**
D10S1656	149	126.09	-	-	-	**-**	0.0	0.0	OAT
D10S217	158	129.54	-	-	-	**-**	0.2	0.2	DOCK1_FOX12
D10S1655	162	130.85	1.52	0.065	1.65	**0.050**	-	-	PTPRE_MGMT
D10S1248	171	130.98	-	-	-	**-**	0.0	0.4	MKI67_MGMT
D10S169	173	132.41	1.30	0.098	2.51	**0.006**	-	-	GLRX3_TCERG1L
D10S1770	169	132.57	2.27	**0.012**	2.69	**0.004**	-	-	GLRX3_TCERG1L
D10S212	171	134.29	1.66	**0.048**	2.42	**0.008**	3.78	1.64	INPP5A
D10S1711	171	135.05	0.90	0.183	2.52	**0.006**	-	-	PAOX_MTG1

**Figure 1:**
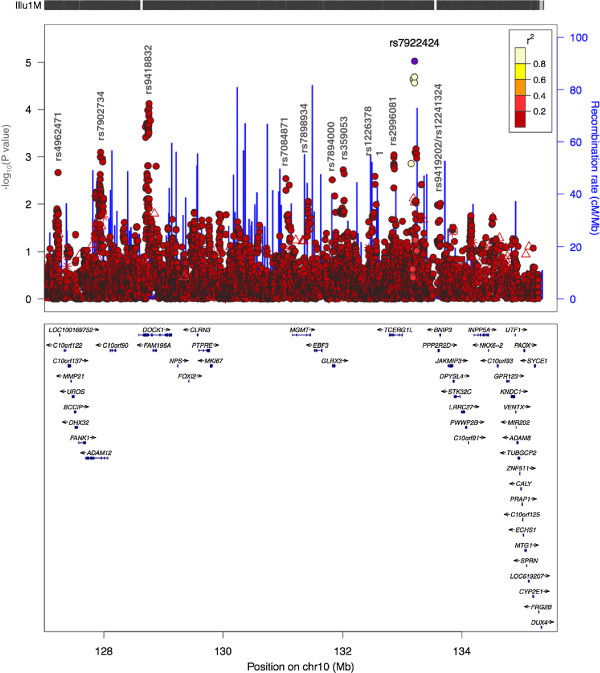
**LocusZoom plots of association for the region spanning 127 Mb to qter in the Raine study.** Pairwise values of linkage disequilibrium with the top SNP (rs7922424; in purple) were calculated using the HapMap CEU population. The top SNP in other genomic regions have been labeled on the plot.

For association mapping, genotype data from the region 127 Mb to 135.35 Mb on chromosome 10 was extracted from Illumina 660 W-Quad BeadChip data in 831 individuals (256 cases and 575 controls) from The Raine Study for whom full covariate data were available [11]. SNP locations on this Illumina chip are provided as NCBI Build 36 base pair locations. Data were available for 10,185 SNPs (2,270 genotyped, 7,915 by imputation). Logistic regression analysis under an additive model, controlling for population substructure using 2 PCs as covariates, provided evidence for several regions of association across the region 127 Mb to qter, with varying degrees of statistical support (Figure 1; Additional file 1: Table S3). In summary, individual top SNPs within genes/regions (P < 10^-3^) are: rs7902734 intronic in *ADAM12* at 127.94 Mb (P = 8.04 × 10^-4^); rs9418832 intronic in *DOCK1* at 128.76 Mb (P = 7.48 × 10^-5^); rs2996081 intronic in *TCERG1L* at 132.86 Mb (P = 9.15 × 10^-4^) and rs7922424 intergenic between *TCERG1L* and *PPP2R2D* at 133.21 Mb (P = 9.47 × 10^-6^). Association signals across these genes/regions were robust to adjustment for independent environmental/clinical covariates (Additional file 1: Table S4).

### Testing for independent effects in genes across the chromosome 10q26 region

**Figure 2:**
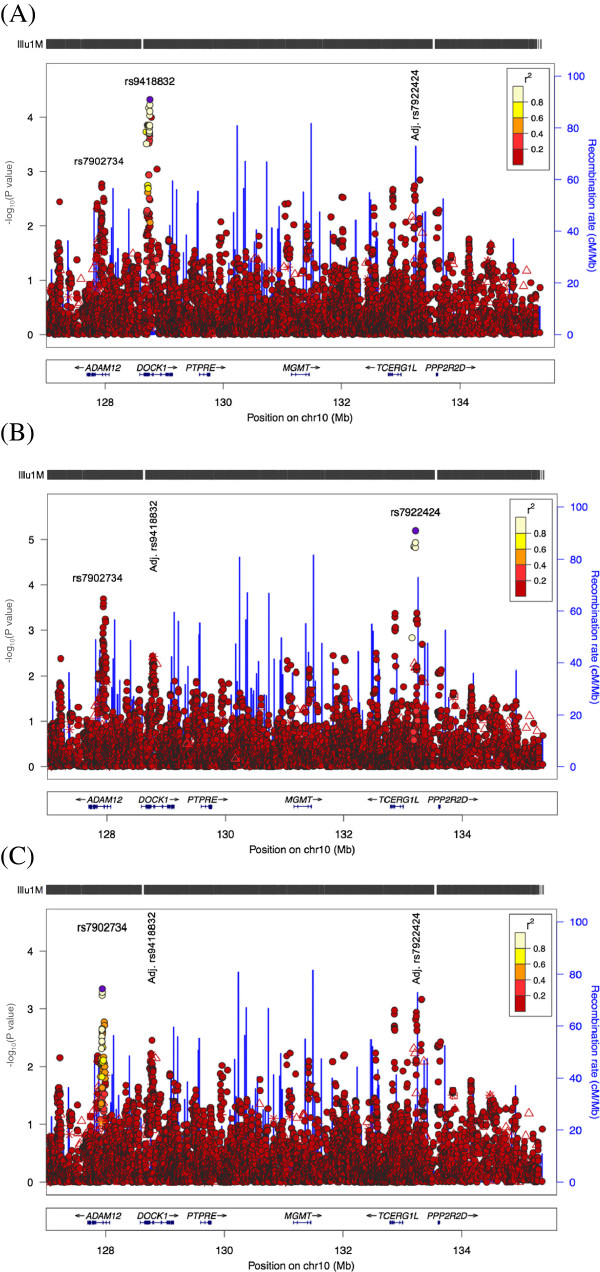
**LocusZoom plots of association adjusted for the top two SNPs, (A) rs7922424, (B) rs9418832 and (C) rs7922424 and rs9418832.** Significance is retained at each SNP after adjusting the analysis for the other. Pairwise values of linkage disequilibrium with the top SNP (in purple) were calculated using the HapMap CEU population.

To evaluate whether the putative regions of association across the 127 Mb to qter region on chromosome 10q26 represented independent signals we repeated the single-point logistic regression analysis adjusting for the most significantly associated SNP (i.e. rs7922424; Figure 2). Upon adjusting for rs7922424 (Figure 2A), the significance at neighbouring SNPs within this intergenic region was lost indicating there is a single main effect across the *TCERG1L*_*PPP2R2D* intergenic region. However, improved significance was observed at *DOCK1* (top SNP rs9418832; P = 4.77 × 10^-5^), suggesting that these genes/regions may be independently associated with OM. All other signals were at P > 10^-3^ after adjusting for rs7922424.

Given evidence of an independent effect at rs9418832 we also adjusted for this SNP (Figure 2B). When adjusting for rs9418832 significance was lost across the *DOCK1* region, again indicating a single main effect within this region, whilst improved significance was observed at rs7922424 (P = 6.47 × 10^-6^), *ADAM12* (top SNP rs7902734; P = 2.05 × 10^-4^) and *TCERG1L* (top SNP rs2996081; P = 4.26 × 10^-4^). The most significant association after adjusting for both rs7922424 and rs9418832 was observed at rs7902734 in *ADAM12* (Figure 2C; P = 4.52 × 10^-4^), indicating that *ADAM12* may also be independently associated with OM.

To formally test for independent effects between *ADAM12*, *DOCK1* and the *TCERG1L_PPP2R2D* intergenic region, we used forward stepwise logistic regression modelling. When comparing models in which the top SNPs are added to each of the other top SNPs (i.e. alternative 2-SNP model compared to the null 1-SNP model; Table 4) results show that they all add independent effects to each other (P_LRT_ ≤ 0.001). Comparison of models containing all three SNPs compared to a null model containing only 2 of the top SNPs confirms these SNPs all add independent effects to each other (*P* ≤ 4.37 × 10^-4^). Overall, the data suggest that variants in the intergenic region between *TCERG1L* and *PPP2R2D*, which lies at 133.2 Mb directly under the peak of linkage (Table 3), as well as in *DOCK1* (128.8 Mb) and *ADAM12* (127.9 Mb), may contribute independently to susceptibility to OM. Therefore, we took these genes/regions forward in evaluating further evidence for the putative etiological gene(s) for OM under the peak of linkage on chromosome 10q26.

**Table 4:** Likelihood ratio tests (LRT) to determine independent effects across the 10q26.3 region

**Null model**	**Alternative model**^**a**^	**LRT χ**^**2**^	**P**_**LRT**_**-value**
**Adding *ADAM12 *** **SNP**			
DOCK1/rs9418832	rs9418832 + **rs7902734**	13.89	1.94 × 10^-4^
Intergenic/rs7922424	rs7922424 + **rs7902734**	9.88	0.0017
rs9418832 + rs7922424	rs9418832 + rs7922424 + **rs7902734**	12.37	4.37 × 10^-4^
**Adding *DOCK1 *** **SNP**			
ADAM12/rs7902734	rs7902734 + **rs9418832**	18.12	2.07 × 10^-5^
Intergenic/rs7922424	rs7922424 + **rs9418832**	16.31	5.39 × 10^-5^
rs7902734 + rs7922424	rs7902734 + rs7922424 + **rs9418832**	18.80	1.45 × 10^-5^
**Adding *TCERG1L_PPP2R2D *** **intergenic SNP**			
ADAM12/rs7902734	rs7902734 + **rs7922424**	17.92	2.31 × 10^-5^
DOCK1/rs9418832	rs9418832 + **rs7922424**	20.11	7.30 × 10^-6^
rs7902734 + rs9418832	rs7902734 + rs9418832 + **rs7922424**	18.60	1.61 × 10^-5^

^a^marker added to the null model in the alternative model is highlighted in bold.

### Expression analysis to support putative functional genes

**Figure 3:**
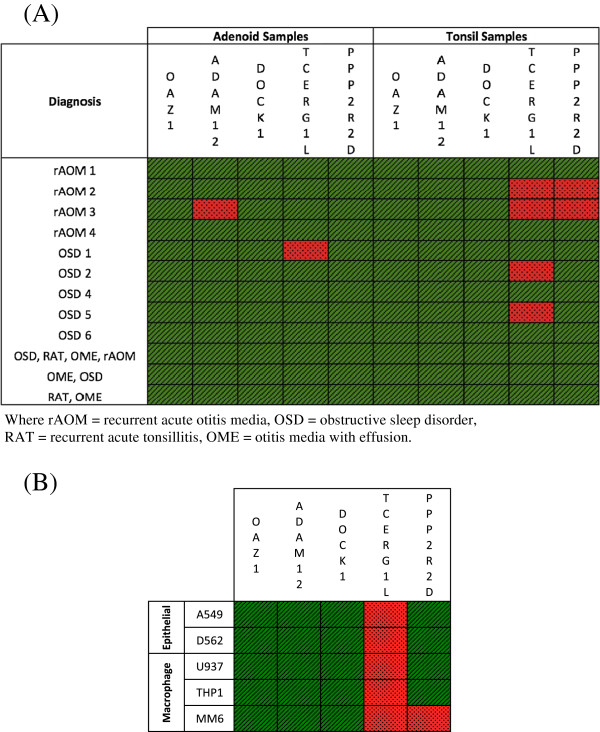
**RT-PCR expression of various candidate genes in (A) adenoids and tonsils of differing phenotypes and (B) in epithelial and macrophage cell lines.** OAZ1 is the housekeeping gene. A green diagonally striped box indicates expression was observed whilst a red dotted box indicates no expression was observed.

Information on the expression pattern of several genes in the 10q26 region is currently limited. Therefore, another way to evaluate potential candidacy of disease associated genes is to determine whether they are expressed in relevant tissue sites *ex vivo*, or in model culture systems *in vitro*. To address the former, we looked at expression of genes in paired adenoid and tonsil tissue from OM cases (rAOM, OME) compared to controls diagnosed with obstructive sleep disorder (OSD) or recurrent acute tonsillitis (RAT) with no concurrent OM history. The role of adenoids in OM pathogenesis is supported by a systematic review showing that adenoidectomy significantly improves resolution of MEE in OM [38] whereas tonsillectomy alone does not [39]. Figure 3A summarises the RT-PCR expression data across adenoid and tonsil samples and shows that *ADAM12*, *DOCK1*, *TCERG1L* and *PPP2R2D* are, for the most part, expressed in all adenoid samples irrespective of OM or OSD diagnosis. In tonsil samples, *ADAM12* and *DOCK1* are again expressed in all samples irrespective of diagnosis. However, *TCERG1L* and *PPP2R2D* expression is less consistent in tonsils with at least two samples showing no expression.

We also looked at epithelial (A549 & Detroit 562) and macrophage (U937, THP-1 & MM6) cell lines (Figure 3B). Again, *ADAM12* and *DOCK1* appear to be expressed in all five cell lines whilst *PPP2R2D* was seen in all cell lines except for MM6. Interestingly, *TCERG1L* was not expressed in epithelial or macrophage cell lines. *TCERG1L* expression was also not detected in any cell line following bacterial challenge with two common otopathogens (NTHi & SP; data not shown), suggesting that any role for this gene in OM would be independent of macrophage or epithelial cell inflammatory responses to otopathogens. It should be noted that this expression data is only qualitative. It is likely that quantitative RT-PCR data would add further information regarding expression levels at these candidates.

### In silico comparative genomics analysis of the TCERG1L to PPP2R2D region

While statistical and expression analyses do not formally exclude any of the genes showing association under the chromosome 10q26 linkage peak with OM, the statistical analyses favour the interval *TCERG1L* to *PPP2R2D* (Additional file 2: Figure S1)*,* which lies directly under the peak of linkage for OM at chromosome 10q26, as the most likely candidate region carrying the etiological variants associated with OM susceptibility. Since the peak of association lies in the intergenic region between these two genes, we carried out an *in silico* analysis to determine whether the associated SNPs in this region lie in highly conserved non-coding sequences that could contain regulatory elements (Additional file 2: Figure S2). Of the 9 associated SNPs (P < 0.001) within the intergenic *TCERG1L* to *PPP2R2D* interval, three are located within highly conserved, non-coding regions (nucleotide conservation level (NCL) ≥ 0.6); rs7037834 (NCL = 0.61), rs7914323 (NCL = 0.61) and rs11813611 (NCL = 0.67). However, rs11813611 lies within a long interspersed nucleotide element (LINEs) repeat region and is unlikely to be functionally relevant. The top SNP, rs7922424, does not lie in a conserved non-coding region but is in strong LD with rs7087384 (r^2^ = 0.97) and rs7914323 (r^2^ = 1.00), both of which do fall in conserved non-coding regions (Additional file 2: Figure S3).

We also looked for the presence of transcription factor binding sites (TFBS) that may be co-located with rs7922424 (top SNP), rs7087384 or rs7914232 (within CNS regions). Results (Additional file 1: Table S5) indicate that rs7922424 lies within the consensus sequence for the Upstream Stimulatory Factor-1 (USF1) transcription factor with the binding site abolished in the presence of the minor disease associated (A) allele. On the other hand, the minor disease associated allele (T) at rs7914323 potentially introduces a USF1 binding site. For rs7087384, the minor disease associated (A) allele disrupts a putative CREB/CREBP1 transcription factor binding site.

In addition to TFBS, we looked at the region containing rs7922424, rs7087384 and rs7914323 to determine whether these SNPs fall in regions identified as a CpG island and as such could disrupt biologically relevant DNA methylation at CpG motifs. Results from CpG Plot and CpG Island Searcher indicate that none of these polymorphisms falls at or near a CpG island (data not shown).

## Discussion

Genome-wide linkage analyses have highlighted five regions containing OM disease susceptibility loci on chromosomes 3p25.3, 10q22.3, 10q26.3, 17q12, and 19q13.43 [6-8]. Here, using a Western Australian cohort of children diagnosed with recurrent OM, we found evidence of replication of linkage at 10q26.3. We also found borderline evidence to support a region of linkage at 10q22.3 but did not find evidence for linkage at 3p25.3, 17q12, or 19q13.43, or for any region after conditioning on 10q26.3. Therefore, we focused attention on mapping the genes under the chromosome 10q26.3 linkage peak.

To identify the etiological gene/variant under the 10q26.3 peak, we performed fine-mapping using directed analysis of 2,270 genotyped SNPs (10,185 after imputation) spanning this region available on 256 cases and 575 controls within the Raine Study cohort. We focused our search in the region 127 Mb to qter, and found statistical support for variants in several genes/regions across this region contributing to the linkage peak. This is not an unusual phenomenon in complex diseases, where previous studies have also highlighted multiple genes contributing to peaks of linkage [40]. In this case, for the region of linkage at 10q26.3, at least 49 genes lie within the interval, a number of which could be considered as potential functional candidates for OM susceptibility. Statistical support was observed for independent effects of SNPs at *ADAM12*, *DOCK1* and the intergenic region between *TCERG1L* and *PPP2R2D*.

The *ADAM12* (A Disintegrin and Metalloproteinase domain 12) gene is a member of the disintegrin and metalloproteinase (ADAM) family of proteins. This gene has been implicated in the epidermal growth factor receptor (EGFR) signalling pathway [41], which is upregulated in human middle ear epithelial cells in response to tobacco smoke exposure [42]. The ADAM12 protein also interacts with the muscle-specific α-actinin-2 protein, with function centred on myoblast/muscle development showing increased expression during muscle generation [43]. The DOCK1 (Dedicator of cytokinesis 1) protein has roles in phagocytosis of apoptotic cells in concert with ELMO1 during Rac signalling and cellular migration [44,45]. The *DOCK1* gene also has a role during embryogenesis in muscle development with knock-out mouse mutants having decreased skeletal and respiratory muscle tissues [46]. This suggests the apoptotic function of *DOCK1* could have a subtle role in the cell death of inflammatory factors and in apoptosis of mucous cells after an immune response to OM, whilst the ADAM12 protein interacts with pathways that could influence expression of inflammatory mediators within the middle ear.

The strongest statistical support for association was in the *TCERG1L* to *PPP2R2D* intergenic region, directly below the peak of linkage on chromosome 10q26.3. Very little is known about the function of the transcription elongation regulator like protein (*TCERG1L*) gene. In recent GWAS studies, variants at or near *TCERG1L* have been associated with fasting insulin, insulin resistance [47] and attention deficit disorder [48]. Hypermethylation of the *TCERG1L* promoter region leading to gene silencing has also been observed in colon cancer [49]. *TCERG1L* expression is documented [50] in a variety of tissues, including the brain, lung and eye. In this study, we have also demonstrated that *TCERG1L* is expressed in adenoids but not in macrophage or epithelial cell lines, either with or without otopathogen infection. Whilst expression in these cells may have been downregulated during immortalization, analogous to downregulation of this gene in cancer cells [49], the data suggest that any role *TCERG1L* may play in OM susceptibility is unlikely to occur through the innate inflammatory response to otopathogens. On balance, *TCERG1L* does not appear to be a strong candidate for OM susceptibility.

In contrast, *PPP2R2D* is a particularly interesting candidate gene for OM. *PPP2R2D* is a member of the B family of regulatory subunits of the protein phosphatase 2A (PP2A) and is widely expressed at the protein level in the brain, heart, placenta, skeletal muscle, testis and thymus [51]. This protein has a role as a modulator of the TGF-β/Activin/Nodal pathway [52], where knockdown of the protein was shown to increase nuclear accumulation and phosphorylation of Smad2. This involvement with Smad2 is of specific interest, as this gene and others in the TGFb pathway have previously been highlighted as candidate susceptibility genes for OM within the WAFSOM cohort [9]. Furthermore, a GWAS carried out in the Raine Study highlighted at least five other members of the TGFb pathway (*BMP5*, *GALNT13, NELL1, TGFB3* and *BPIFA1*) as candidates for OM susceptibility [11].

The strongest signal for association in our study lay within the intergenic region between the *TCERG1L* and *PPP2R2D* genes. Many association signals in complex diseases have been found to lie within intergenic regions [40], leading to a search for potential regulatory functions within those regions. Whilst the top SNP (rs7922424) in the intergenic region does not itself lie within a conserved non-coding region, it is in strong LD with rs7087384 and rs7914323, which do lie in highly conserved non-coding regions. In addition, the minor disease associated alleles at rs7922424 and rs7914323 alter putative binding sites for the upstream stimulatory factor 1 (USF1). The USF1 transcription factor is a member of the helix-loop-helix leucine zipper family and is ubiquitously expressed in a variety of cells [53]. The role of this protein is widespread, ranging from roles in embryonic development [54] to promoters for a number of activity-induced genes within neuronal nuclei [55] and as transcription factors that regulate cell-type dependent cellular proliferation [56]. However, our data favours rs7914323 as the most likely regulatory polymorphism in this region. Not only does this SNP lie within a conserved, non-coding region that could harbor regulatory elements, but our analysis shows that the presence of the minor allele potentially eliminates a TFBS for the cAMP response element binding (CREB) transcription factor and its binding protein (CREBP or CBP). This is interesting in the context of OM as the function of CBP has been linked to the TGFb pathway via the recruitment of EVI1 [57], which is mutated in a mouse model of OM [58], although the *EVI1* gene has not been associated with human susceptibility to OM to date [9,59].

Overall, analysis of conserved, non-coding regions and putative TFBS sites indicate a number of regulatory elements that lie within this intergenic region that can potentially be influenced by polymorphisms associated with OM susceptibility. It is not possible to determine from these data which gene these regulatory elements may influence. However, the TFBS consensus sequences disrupted by these polymorphisms all lie on the forward strand, upstream of the *PPP2R2D* promoter. The *TCERG1L* gene on the other hand is encoded on the reverse strand. Taken together these observations strengthen the evidence for *PPP2R2D* as the likely gene contributing to linkage at 10q26.3.

## Conclusions

Using the resources available within the family-based WAFSOM cohort the results of our study provide the first replication of linkage to OM susceptibility at chromosome 10q26.3. Subsequent association analysis using data available within the longitudinal Raine Study show that multiple genes could contribute to this linkage peak, however the weight of evidence supports *PPP2R2D* as the more likely functional candidate in this linkage region. It has previously been noted that there is phenotypic heterogeneity between WAFSOM and the Raine Study [11]. The phenotype defined for the Raine Study is biased towards the milder end of the OM spectrum, being based on yearly clinical examinations and parental reports of ≥3 AOM episodes during the first three years of life. The WAFSOM cohort on the other hand is biased towards the more severe end of the OM spectrum, being based on children recommended for grommet insertion. This suggests that *PPP2R2D* plays a role in susceptibility to OM *per se*. In summary our results point to a role for *PPP2R2D,* a TGF-β/Activin/Nodal pathway modulator, as the more likely functional candidate for OM susceptibility at chromosome 10q26.3. This contributes to the growing evidence for a role for the TGFb pathway in susceptibility to this important childhood disease.

## Competing interests

The authors declare that they have no competing interests.

## Authors’ contributions

Conceived and designed the experiments: SEJ JMB MSR. Clinical care and characterization for WAFSOM: SV HLC. WAFSOM recruitment and preparation: ESHS SEJ MSR. Raine Study management: CEP. Adenoid/Tonsil collection and preparation: RT SV HLC. WAFSOM genotyping, cell culture, RNA preparation and RT-PCR expression: MSR. Design and maintenance of in-house genetic database for WAFSOM and bioinformatics CNS pipeline: RWF. Analyzed the data: MSR SEJ JMB. Supervised the work: SEJ JMB CEP. Wrote the paper: MSR SEJ JMB. Reviewed the manuscript. All authors read and approved the final manuscript.

## Pre-publication history

The pre-publication history for this paper can be accessed here:

http://www.biomedcentral.com/1471-2350/15/18/prepub

## Supplementary Material

Additional file 1: Table S1 Microsatellite markers and their respective primer sequences used for replication of previously reported linkage regions. **Table S2.** Forward and reverse primer sequences for qualitative RT-PCR expression of four chromosome 10 candidate genes. **Table S3.** Results for SNPs P < 10^-3^ in the Raine Study across the region of chromosome 10 from 127 Mb to qter. **Table S4.** Raine Study results for the 10q26.3 region showing respective top SNPs for various genes/regions after adjusting for known environmental covariates using ProbABEL. **Table S5.** Details of transcription factor binding sites for top SNPs across the *TCERG1L/PPP2R2D* gene region. A transcription factor is shown only when a change in allele adds or removes a transcription factor binding site. Click here for file

Additional file 2: Figure S1 LocusZoom plot of association (Figure 1) focused on the *TCERG1L*/*PPP2R2D* intergenic region. Pairwise values of LD with the top SNP (rs7922424; in purple) were calculated using the HapMap CEU population. **Figure S2.** Conserved non-coding sequence plot of the *TCERG1L*/*PPP2R2D* intergenic region containing the 9 associated SNPs (P < 0.0001). The top SNP (rs7922424) is annotated in orange, with SNPs P < 0.0001 in green and additional SNPs (P < 0.001) annotated in purple. Colours indicate the following regions; repeats (blue), genes (brown) and coding sequence (red). The three SNPs of interest are highlighted by bold text. Species are human (H), mouse (M), and rat (R). **Figure S3.** Pairwise LD between the 9 associated SNPs (P < 0.0001) in the Raine Study cohort, where (A) is the D’ value and (B) is the r^2^ value. For both D’ and r^2^, LD measures are indicated at the matrix intercept between two markers and are indicative of a decimal value. A square with no value indicated at the intercept equates to a value of 1.00 (or complete LD). For D’ measures, red and shades of red indicate a higher degree of confidence (i.e. LOD ≥2.0). Click here for file
