# Supplementary material for: Genetic and functional evidence for a locus controlling otitis media at chromosome 10q26.3
Source: BMC Med Genet. 2014 Feb 6;15:18. doi: 10.1186/1471-2350-15-18 (PMC3926687; doi:10.1186/1471-2350-15-18)
Supplement: Additional file 1: Table S1 — Microsatellite markers and their respective primer sequences used for replication of previously reported linkage regions. Table S2. Forward and reverse primer sequences for qualitative RT-PCR expression of four chromosome 10 candidate genes. Table S3. Results for SNPs P < 10-3 in the Raine Study across the region of chromosome 10 from 127 Mb to qter. Table S4. Raine Study results for the 10q26.3 region showing respective top SNPs for various genes/regions after adjusting for known environmental covariates using ProbABEL. Table S5. Details of transcription factor binding sites for top SNPs across the TCERG1L/PPP2R2D gene region. A transcription factor is shown only when a change in allele adds or removes a transcription factor binding site. [file 1471-2350-15-18-S1.pdf]

**Table S1:** Microsatellite markers and their respective primer sequences used for replication of previously reported linkage regions.

| Marker Name | Labelled Primer Sequence (5' to 3') | Unlabelled Primer Sequence (5' to 3') | Het[a] | N[0] Alleles[b] |
|---|---|---|---|---|
| ***Chromosome 3p25.3*** | | | | |
| D3S1259[c] | GCTGGACTATATTTGAAACTCAT | TTTCAGTGAGCCAAGATCGT | 0.83 | 14 |
| D3S1263 | CTGTTGACCCATTGATACCC | TAAAATCACAGCAGGGGTTC | 0.86 | 17 |
| D3S1304 | TTCGCTCTTTGATAGGC | ATTTCATTTGTAATTTACTAGCAG | 0.79 | 13 |
| D3S1597 | AGTACAAATACACACAAATGTCTC | GCAAATCGTTCATTGCT | 0.80 | 10 |
| D3S3691 | TCTCAGCAATAGCAAACATCAGG | TTGAAACCAGGGTGACAAATACATC | 0.67 | 7 |
| ***Chromosome 10q22.3*** | | | | |
| D10S1677 | ACTGCTCAGCATATTTTATGGC | AGTTCATGGCTCCCTCA | 0.70 | 8 |
| D10S1730 | GTGCAGCCACTGTTGAGAG | AAGTTTGAGAACCACTGGTCTATC | 0.83 | 14 |
| D10S201 | AGCTCATGGGATGGAAGCAT | AGCTAAAAGGCTGCTGGAGA | 0.82 | 14 |
| D10S206 | CCAAAACGTGGTCACAGATA | GGATCCATGAAAATGCACCT | 0.56 | 8 |
| ***Chromosome 10q26.3*** | | | | |
| D10S1655 | TGCCAGACTCCATGATGGTATGAAC | TAACAAACATCTGAGCACAGCCCC | 0.67 | 9 |
| D10S169 | GATCTGTGACTGCCTTCCT | AAGAGGAGGAGTCCATTCAG | 0.73 | 10 |
| D10S1770 | CATGAGATTTGAGTGGGG | AACAGGCTGTGGCCTC | 0.82 | 12 |
| D10S212[c] | GAAGTAAAGCAAGTTCTATCCACG | TCTGTGTACGTTGAAAATCCC | 0.71 | 7 |
| D10S1711 | CTATGATGCTGAGGGCAGTG | ATGCTACCATGCCTGGCTAC | 0.70 | 7 |
| ***Chromosome 17q12*** | | | | |
| D17S1293 | TGGAGGCTAGGAGTTTTCCT | GGAGGCAGTGAGTTGTGATT | 0.83 | 11 |
| D17S927 | TGTCTGACATGACACCCCT | TCCAACCTGAAGGCCAGT | 0.72 | 8 |
| D17S933 | ACTCACTGGGGTCCTGG | TGTGGTTTCCTTATAGACTGTAGA | 0.82 | 9 |
| D17S946 | ACAGTCTATCAAGCAGAAAAATCCT | TGCCGTGCCAGAGAGA | 0.80 | 10 |
| ***Chromosome 19q13.43*** | | | | |
| D19S210 | TCACACTCACTGGTCTCTCA | GGTGTGCCTGTGTGTAAAAG | 0.73 | 7 |
| D19S214[c] | AGTCACAATGGAGACACATACATAC | CAACTGGCTTTATCCCACTC | 0.63 | 14 |
| D19S254 | TTCTGAGCACAACCAATCCT | GACTCCGTATTAAAAAAAAAAG | 0.77 | 11 |
| D19S572 | ATTTGGGTGTGCTGACACG | GGGCCACATCCTATCCCC | 0.80 | 11 |
| D19S887 | TATCCAATGCCACAGAAAA | AAGGTTTGCTTGTTTGGGT | 0.74 | 9 |

[a] Heterozygosity; [b] Number of alleles seen in WAFSOM cohort; [c] Markers with significant linkage in the GWLS reported by Daly *et al.* [6]

**Table S2:** Forward and reverse primer sequences for qualitative RT-PCR expression of four chromosome 10 candidate genes.

| Gene | Forward Primer Sequence (5' to 3') | Reverse Primer Sequence (5' to 3') |
|---|---|---|
| *ADAM12* | AGGAATTCTGGTGACCATCC | TCGGTGGGTAGGAATCTGG |
| *DOCK12* | CTGGTGGTTAGAGATGAAGATGG | TGATGAATTTGGACTCCACAGG |
| *TCERG1L* | AAATTGCTGCTAGCCAAAGAAG | GTCCGTCTCCACCGTGAC |
| *PPP2R2D* | GCACCTTTCAAAGTCATGAACC | CTTCCATCTTCGTCTTTCAGG |

**Table S3.** Results for SNPs $P<10^{-3}$ in the Raine Study across the region of Chromosome 10 from 127Mb to qter.

| CHR | GENE | SNP | Effect Allele | Allele Freq | POSITION | PCA | | CONSEQUENCE |
|---|---|---|---|---|---|---|---|---|
| | | | | | | P-value | OR (95% CI) | |
| 10 | ADAM12 | rs7902734 | A | 0.35 | 127942891 | $8.04x10^{-4}$ | 1.47 (1.17-1.85) | INTRONIC |
| 10 | DOCK1 | rs1538785 | C | 0.90 | 128694878 | $2.33x10^{-4}$ | 0.53 (0.37-0.74) | INTRONIC |
| 10 | DOCK1 | rs4751179 | A | 0.90 | 128699635 | $3.88x10^{-4}$ | 0.53 (0.38-0.75) | INTRONIC |
| 10 | DOCK1 | rs7068941 | A | 0.10 | 128718078 | $1.96x10^{-4}$ | 1.92 (1.36-2.71) | INTRONIC |
| 10 | DOCK1 | rs7079472 | C | 0.91 | 128721593 | $2.03x10^{-4}$ | 0.52 (0.37-0.73) | INTRONIC |
| 10 | DOCK1 | rs2185404 | A | 0.09 | 128723423 | $2.04x10^{-4}$ | 1.92 (1.36-2.72) | INTRONIC |
| 10 | DOCK1 | rs7085407 | C | 0.91 | 128726204 | $2.10x10^{-4}$ | 0.52 (0.37-0.73) | INTRONIC |
| 10 | DOCK1 | rs880272 | A | 0.09 | 128730832 | $2.14x10^{-4}$ | 1.92 (1.36-2.72) | INTRONIC |
| 10 | DOCK1 | rs1538788 | A | 0.10 | 128735637 | $2.30x10^{-4}$ | 1.91 (1.35-2.7) | INTRONIC |
| 10 | DOCK1 | rs7092964 | C | 0.90 | 128737210 | $1.04x10^{-4}$ | 0.51 (0.37-0.72) | INTRONIC |
| 10 | DOCK1 | rs9418726 | G | 0.68 | 128742134 | $3.26x10^{-4}$ | 0.66 (0.52-0.83) | INTRONIC |
| 10 | DOCK1 | rs1953965 | A | 0.10 | 128742319 | $2.68x10^{-4}$ | 1.89 (1.34-2.67) | INTRONIC |
| 10 | DOCK1 | rs7909186 | C | 0.32 | 128742746 | $3.46x10^{-4}$ | 1.52 (1.21-1.91) | INTRONIC |
| 10 | DOCK1 | rs7923722 | C | 0.90 | 128744069 | $2.97x10^{-4}$ | 0.53 (0.38-0.75) | INTRONIC |
| 10 | DOCK1 | rs1417153 | A | 0.09 | 128746542 | $2.26x10^{-4}$ | 1.92 (1.36-2.72) | INTRONIC |
| 10 | DOCK1 | rs7918376 | C | 0.90 | 128748249 | $2.92x10^{-4}$ | 0.53 (0.38-0.75) | INTRONIC |
| 10 | DOCK1 | rs4751519 | C | 0.90 | 128748514 | $2.92x10^{-4}$ | 0.53 (0.38-0.75) | INTRONIC |
| 10 | DOCK1 | rs4751520 | C | 0.10 | 128748549 | $2.87x10^{-4}$ | 1.89 (1.34-2.66) | INTRONIC |
| 10 | DOCK1 | rs4751533 | A | 0.10 | 128751289 | $2.65x10^{-4}$ | 1.9 (1.34-2.68) | INTRONIC |
| 10 | DOCK1 | rs9418822 | A | 0.32 | 128752085 | $3.06x10^{-4}$ | 1.52 (1.21-1.91) | INTRONIC |
| 10 | DOCK1 | rs4635002 | A | 0.10 | 128752669 | $2.21x10^{-4}$ | 1.91 (1.36-2.69) | INTRONIC |
| 10 | DOCK1 | rs6482987 | A | 0.10 | 128753770 | $2.06x10^{-4}$ | 1.92 (1.36-2.72) | INTRONIC |
| 10 | DOCK1 | rs7917277 | C | 0.90 | 128754664 | $1.38x10^{-4}$ | 0.51 (0.36-0.72) | INTRONIC |
| 10 | DOCK1 | rs9418829 | A | 0.30 | 128755590 | $3.12x10^{-4}$ | 1.55 (1.22-1.96) | INTRONIC |
| 10 | DOCK1 | rs1417154 | A | 0.10 | 128756210 | $1.18x10^{-4}$ | 2 (1.4-2.84) | INTRONIC |
| 10 | DOCK1 | rs9418737 | A | 0.10 | 128757840 | $9.21x10^{-5}$ | 2.04 (1.43-2.91) | INTRONIC |
| 10 | DOCK1 | rs9418832 | A | 0.10 | 128757891 | $7.48x10^{-5}$ | 2.06 (1.44-2.95) | INTRONIC |
| 10 | DOCK1 | rs7091697 | A | 0.56 | 128783565 | $1.71x10^{-4}$ | 0.66 (0.53-0.82) | INTRONIC |
| 10 | TCERG1L | rs2996081 | A | 0.11 | 132860626 | $9.15x10^{-4}$ | 0.49 (0.32-0.75) | INTRONIC |
| 10 | | rs12250300 | A | 0.07 | 133183097 | $2.28x10^{-5}$ | 2.34 (1.58-3.47) | INTERGENIC |
| 10 | | rs7087384 | A | 0.07 | 133185799 | $2.28x10^{-5}$ | 2.34 (1.58-3.46) | INTERGENIC |
| 10 | | rs7912988 | G | 0.93 | 133203929 | $2.72x10^{-5}$ | 0.43 (0.29-0.64) | INTERGENIC |
| 10 | | rs7914323 | C | 0.93 | 133204205 | $2.05x10^{-5}$ | 0.42 (0.28-0.63) | INTERGENIC |
| 10 | | rs7922424 | A | 0.07 | 133206442 | $9.47x10^{-6}$ | 2.49 (1.66-3.73) | INTERGENIC |
| 10 | | rs11813611 | C | 0.90 | 133216884 | $8.09x10^{-4}$ | 2 (1.33-3) | INTERGENIC |
| 10 | | rs11018027 | A | 0.10 | 133229112 | $6.78x10^{-4}$ | 0.51 (0.34-0.75) | INTERGENIC |
| 10 | | rs10830030 | A | 0.12 | 133229698 | $8.96x10^{-4}$ | 0.53 (0.36-0.77) | INTERGENIC |
| 10 | | rs10765090 | C | 0.13 | 133238729 | $8.57x10^{-4}$ | 0.55 (0.38-0.78) | INTERGENIC |

OR = odds ratio, 95% CI = 95% confidence intervals. Odds ratios are stated for the effect (disease-associated) allele.

**Table S4:** Raine Study results for the 10q26.3 region showing respective top SNPs for various genes/regions after adjusting for known environmental covariates using ProbABEL.

| CHR | GENE | SNP | Effect Allele | Allele Freq | POSITION | PCA | | PCA + Daycare | | PCA + Allergy | |
|---|---|---|---|---|---|---|---|---|---|---|---|
| | | | | | | P-value | OR (95% CI) | P-value | OR (95% CI) | P-value | OR (95% CI) |
| 10 | ADAM12 | rs7902734 | A | 0.34 | 127942891 | $8.04 \times 10^{-4}$ | 1.47 (1.17-1.85) | $6.04 \times 10^{-4}$ | 1.49 (1.19-1.87) | $6.93 \times 10^{-4}$ | 1.48 (1.18-1.86) |
| 10 | DOCK1 | rs9418832 | A | 0.10 | 128757891 | $7.48 \times 10^{-5}$ | 2.06 (1.44-2.95) | $8.62 \times 10^{-5}$ | 2.05 (1.43-2.94) | $1.07 \times 10^{-4}$ | 2.04 (1.42-2.92) |
| 10 | TCERG1L | rs2996081 | C | 0.89 | 132860626 | $9.15 \times 10^{-4}$ | 2.04 (1.33-3.13) | $7.00 \times 10^{-4}$ | 2.08 (1.37-3.23) | $9.84 \times 10^{-4}$ | 2.04 (1.33-3.13) |
| 10 | | rs7922424 | A | 0.07 | 133206442 | $9.47 \times 10^{-6}$ | 2.49 (1.66-3.73) | $1.55 \times 10^{-5}$ | 2.45 (1.63-3.67) | 4.96x10-6 | 2.58 (1.72-3.88) |

| CHR | GENE | SNP | Effect Allele | Allele Freq | POSITION | PCA + Other Milk | | PCA + All Covariates | | CONSEQUENCE |
|---|---|---|---|---|---|---|---|---|---|---|
| | | | | | | P-value | OR (95% CI) | P-value | OR (95% CI) | |
| 10 | ADAM12 | rs7902734 | A | 0.34 | 127942891 | $7.35 \times 10^{-4}$ | 1.48 (1.18-1.86) | $5.55 \times 10^{-4}$ | 1.50 (1.19-1.89) | INTRONIC |
| 10 | DOCK1 | rs9418832 | A | 0.10 | 128757891 | $2.82 \times 10^{-5}$ | 2.17 (1.51-3.11) | $4.56 \times 10^{-5}$ | 2.13 (1.48-3.07) | INTRONIC |
| 10 | TCERG1L | rs2996081 | C | 0.89 | 132860626 | $1.01 \times 10^{-3}$ | 2.04 (1.33-3.13) | $9.07 \times 10^{-4}$ | 2.04 (1.33-3.13) | INTRONIC |
| 10 | | rs7922424 | A | 0.07 | 133206442 | $1.27 \times 10^{-5}$ | 2.47 (1.65-3.72) | $1.10 \times 10^{-5}$ | 2.52 (1.67-3.81) | INTERGENIC |

Where OR = odds ratio, 95% CI = 95% confidence intervals. Odds ratios are stated for the effect (disease-associated) allele.

**Table S5:** Details of transcription factor binding sites for top SNPs across the *TCERG1L*/*PPP2R2D* gene region. A transcription factor is shown only when a change in allele adds or removes a particular transcription factor binding site.

| | | **Program** | |
|---|---|---|---|
| **Gene/SNP** | **Allele** | **ALIBABA2** | **MatInspector** |
| Intergenic | G | C-Jun/CREBP1/CPE/C-EBPa | CREB/AP1R |
| rs7087384 | A | - | - |
| Intergenic | C | - | HTEN |
| rs7914323 | T | USF | - |
| Intergenic | G | USF | MYC/USF |
| rs7922424 | A | - | - |

N.B. Major allele listed first.
