# Supplementary material for: Genetic and functional evidence for a locus controlling otitis media at chromosome 10q26.3
Source: BMC Med Genet. 2014 Feb 6;15:18. doi: 10.1186/1471-2350-15-18 (PMC3926687; doi:10.1186/1471-2350-15-18)

Figure S1 : LocusZoom plot of association (Figure 1) focused on the *TCERG1L*/*PPP2R2D* intergenic region. Pairwise values of LD with the top SNP (rs7922424; in purple) were calculated using the HapMap CEU population.

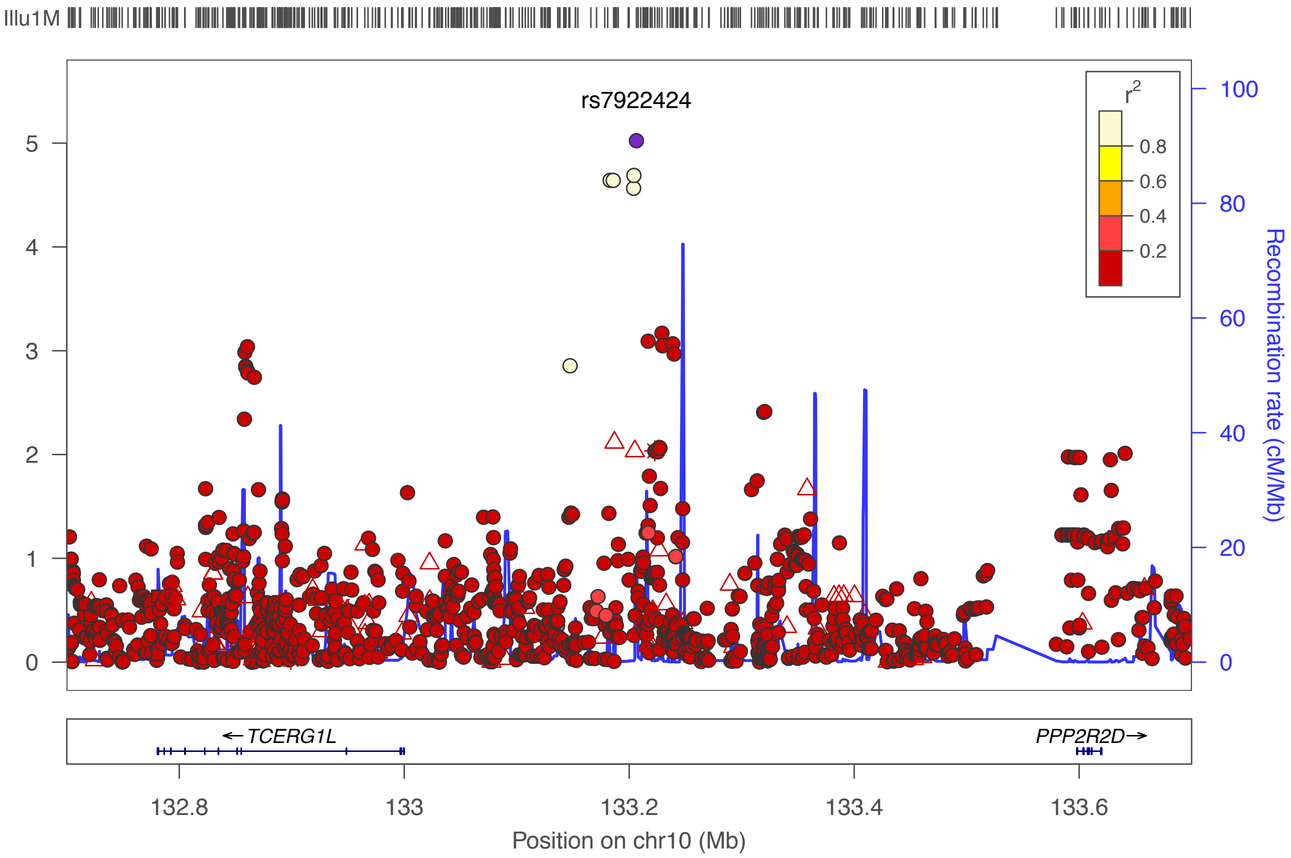

Figure S2. Conserved non-coding sequence plot of the intergenic region containing the 9 top intergenic SNPs between *TCERG1L* and *PPP2R2D*. The top SNP (rs7922424) is annotated in orange, with SNPs P<0.0001 in green and additional SNPs (P<0.001) annotated in purple. Colours indicate the following regions; repeats (blue), genes (brown) and coding sequence (red). The three SNPs of interest are highlighted by bold text. Species are human (H), mouse (M), and rat (R).

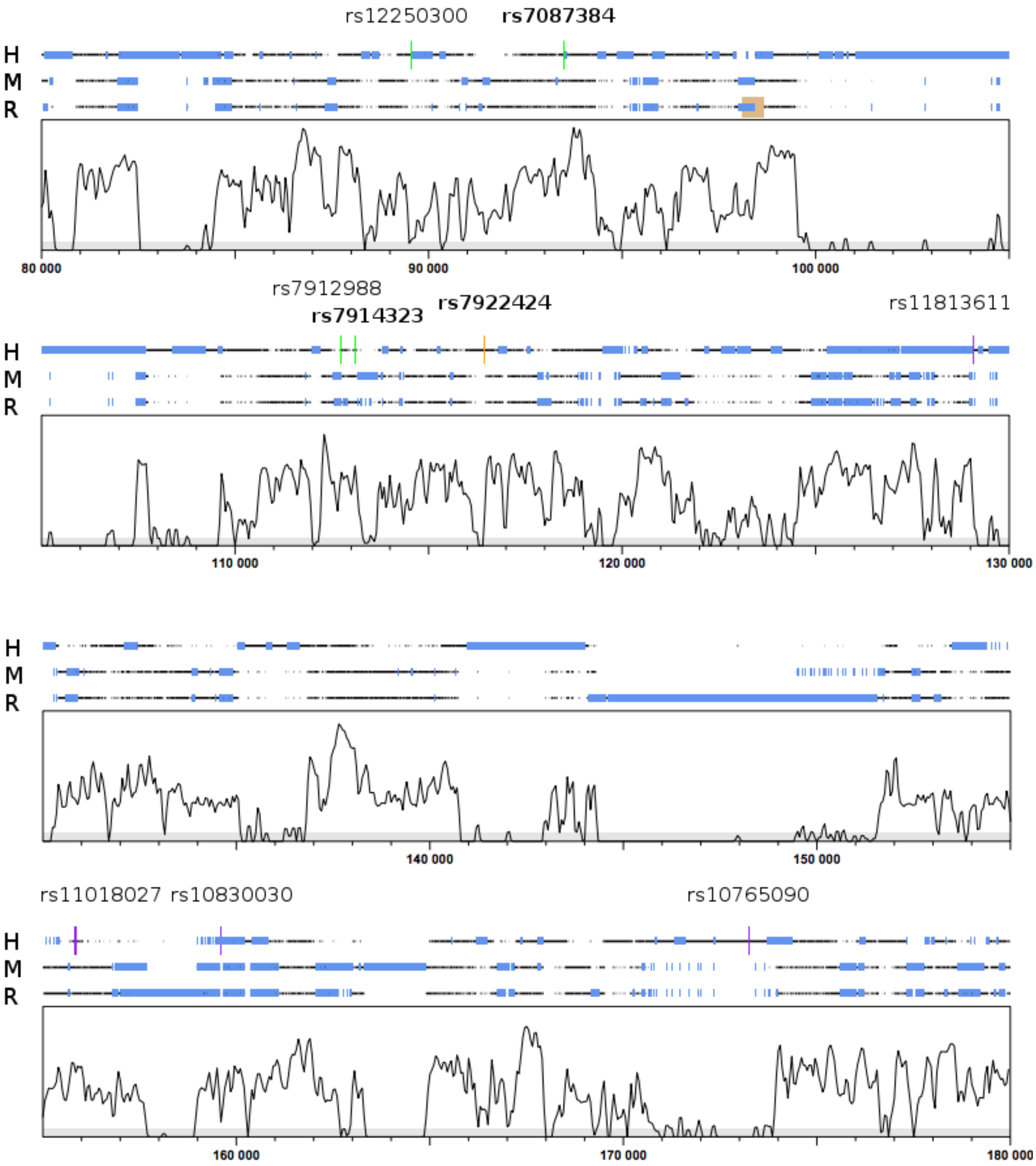

Figure S3. Pairwise LD between the 9 associated SNPs ($P<0.0001$) in the Raine Study cohort, where (A) is the D` value and (B) is the r^2 value. For both D' and r^2, LD measures are indicated at the matrix intercept between two markers and are indicative of a decimal value. A square with no value indicated at the intercept equates to a value of 1.00 (or complete LD). For D' measures, red and shades of red indicate a higher degree of confidence (i.e. LOD ≥2.0).

(A)

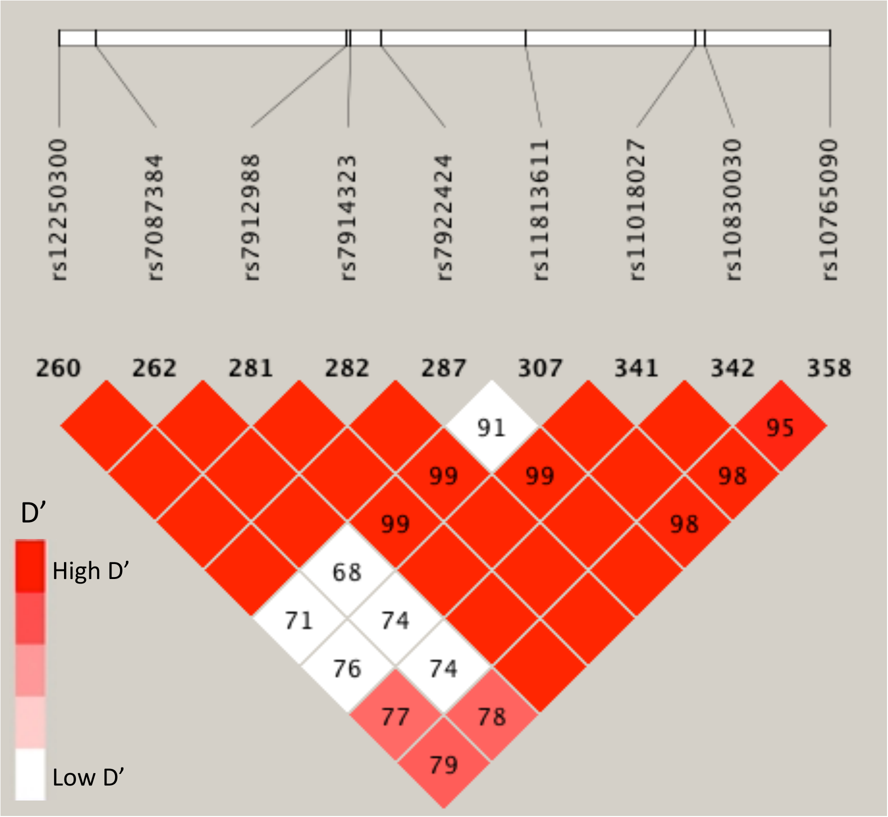

(B)

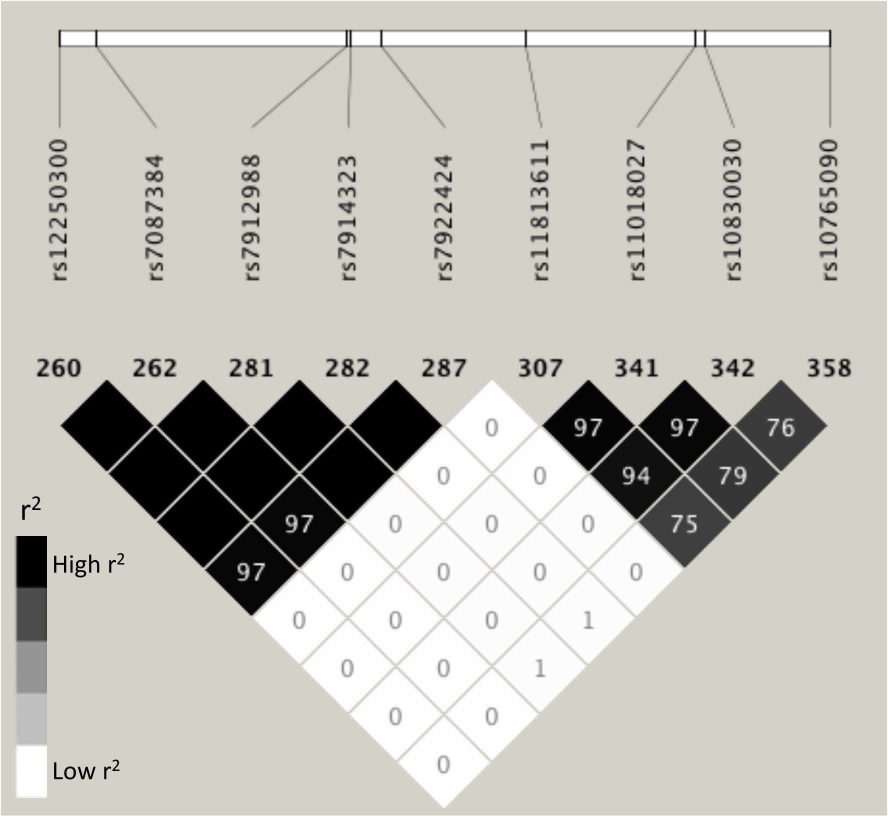

Supplement: Additional file 2: Figure S1 — LocusZoom plot of association (Figure 1) focused on the TCERG1L/PPP2R2D intergenic region. Pairwise values of LD with the top SNP (rs7922424; in purple) were calculated using the HapMap CEU population. Figure S2. Conserved non-coding sequence plot of the TCERG1L/PPP2R2D intergenic region containing the 9 associated SNPs (P < 0.0001). The top SNP (rs7922424) is annotated in orange, with SNPs P < 0.0001 in green and additional SNPs (P < 0.001) annotated in purple. Colours indicate the following regions; repeats (blue), genes (brown) and coding sequence (red). The three SNPs of interest are highlighted by bold text. Species are human (H), mouse (M), and rat (R). Figure S3. Pairwise LD between the 9 associated SNPs (P < 0.0001) in the Raine Study cohort, where (A) is the D’ value and (B) is the r2 value. For both D’ and r2, LD measures are indicated at the matrix intercept between two markers and are indicative of a decimal value. A square with no value indicated at the intercept equates to a value of 1.00 (or complete LD). For D’ measures, red and shades of red indicate a higher degree of confidence (i.e. LOD ≥2.0). [file 1471-2350-15-18-S2.pdf]
